# Supplementary material for: Proteome-wide analysis of cysteine oxidation reveals metabolic sensitivity to redox stress
Source: Nat Commun. 2018 Apr 20;9:1581. doi: 10.1038/s41467-018-04003-3 (PMC5910380; doi:10.1038/s41467-018-04003-3)
Supplement: Supplementary file 1 — Supplementary Information [file 41467_2018_4003_MOESM1_ESM.pdf]

Proteome-wide analysis of cysteine oxidation reveals metabolic sensitivity to redox stress

van der Reest et al.

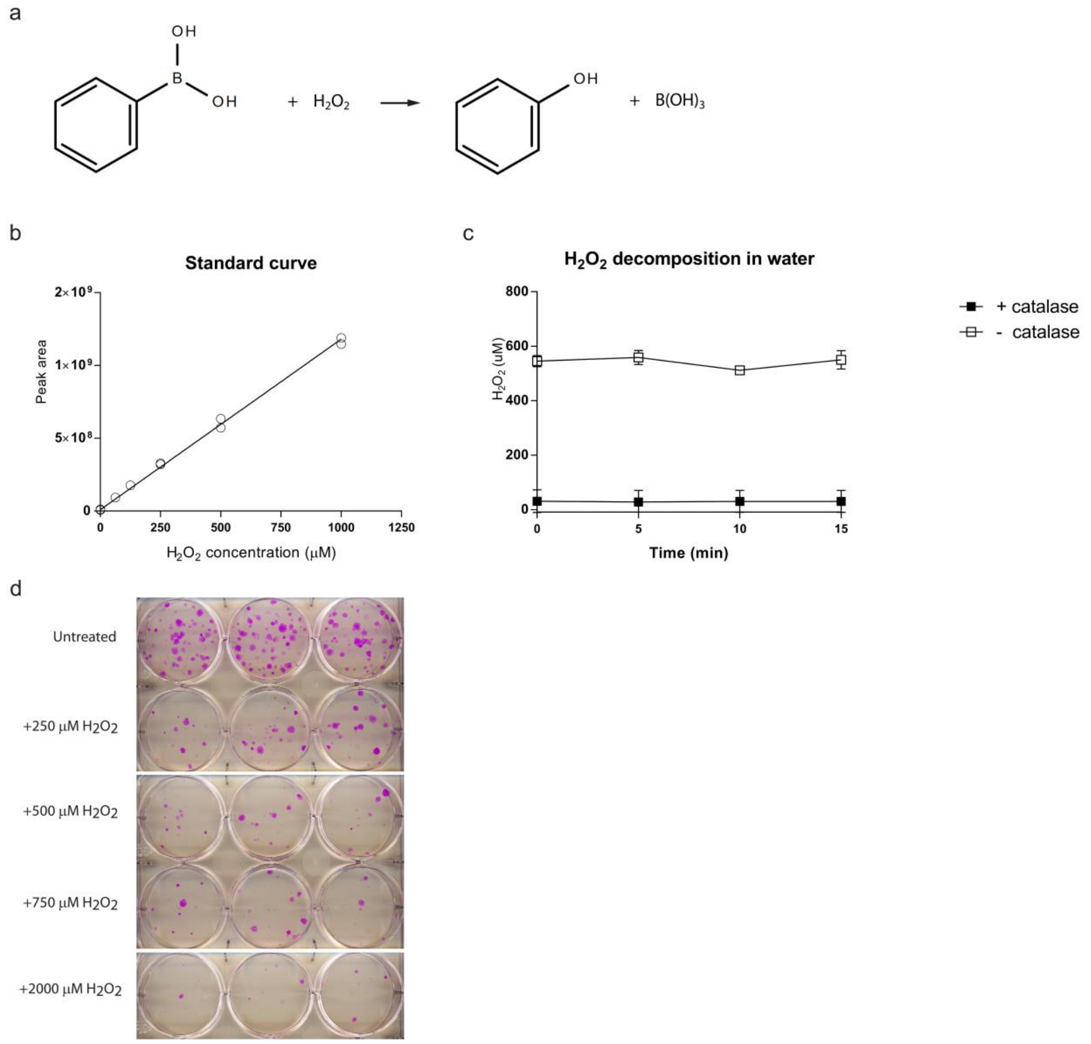

Supplementary Fig. 1. Treatment with hydrogen peroxide causes recoverable oxidative stress in *Fh1*$^{fl/fl}$ cells. (**a**) Chemical reaction of phenylboronate with hydrogen peroxide to yield phenol, which is detected by LC-MS/MS as a probe for hydrogen peroxide. (**b**) Standard curve of the phenylboronate-based hydrogen peroxide quantification assay, showing linearity for the concentration range used. (**c**) Positive (500 µM hydrogen peroxide in water) and negative control (500 µM hydrogen peroxide in water incubated with catalase) for indicated time points. (**d**) Images of colony formation assays after treatment with hydrogen peroxide for 15 minutes at the indicated concentrations. (**b**) Representative graph of 3 independent experiments with duplicate measurements; (**c**) mean (S.D.) of 3 independent experiments with duplicate measurements; (**d**) representative image of 3 independent experiments with triplicate measurements (quantification in Fig. 3c).

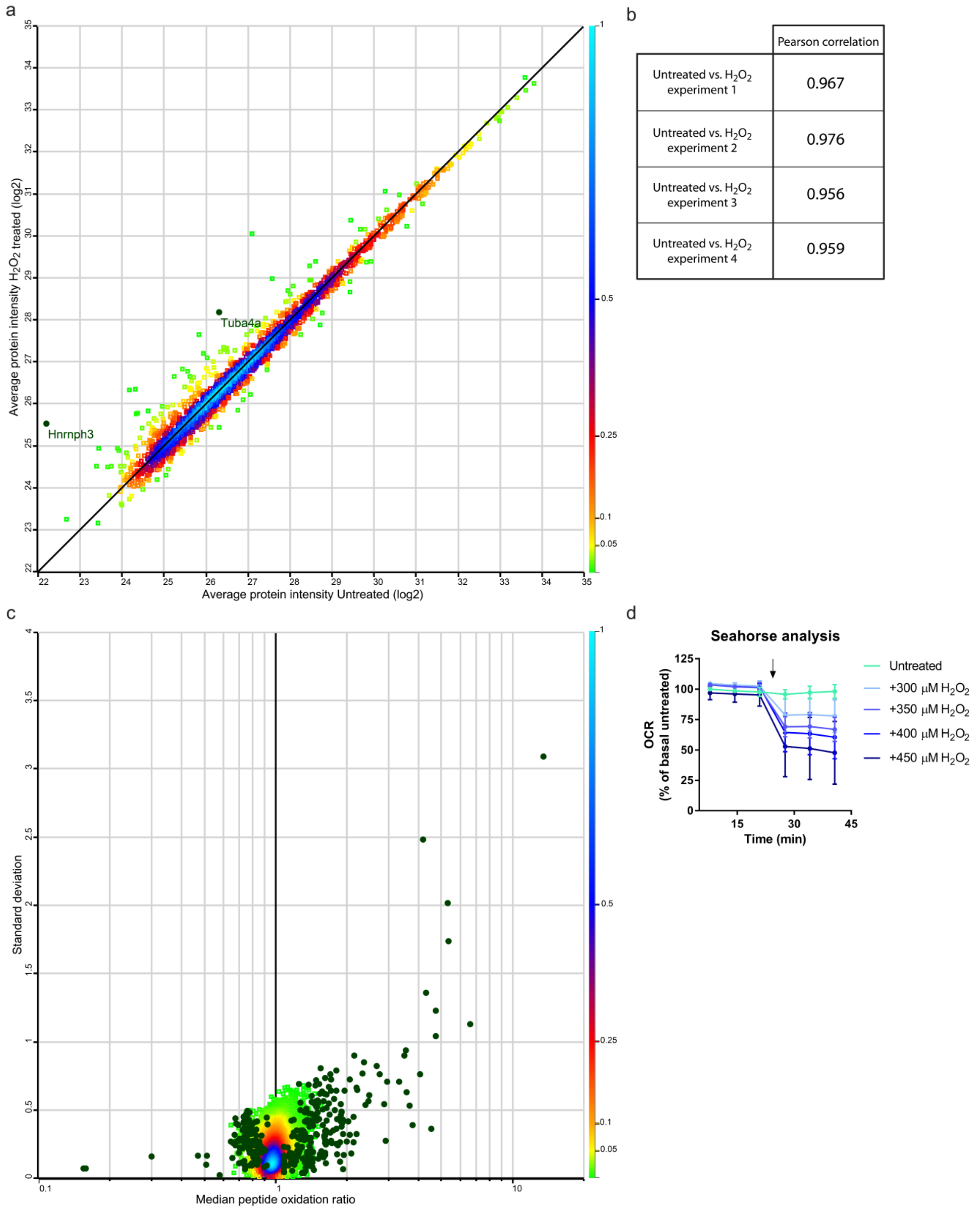

Supplementary Fig. 2. Acute oxidative stress causes oxidation of predominantly metabolic and mitochondrial proteins. (**a**) Density scatterplot displaying average protein intensity in the untreated *Fh1*$^{fl/fl}$ cells versus cells treated with 500 μM hydrogen peroxide for 15 minutes. Every square represents a unique protein; colour scale of the density of the data points in the corresponding region is indicated on the right. Highlighted green circles are proteins with a significantly different

intensity between samples (t-test analysis, FDR 0.05); corresponding gene names are displayed. (**b**) Pearson correlation of the protein intensity in the untreated versus hydrogen peroxide-treated cells for the individual replicate experiments that were averaged in panel a. (**c**) Density scatterplot displaying median peptide oxidation ratios versus standard deviation in *Fh1*$^{fl/fl}$ cells treated with 500 µM hydrogen peroxide for 15 minutes compared to untreated cells. Every square represents a unique peptide; colour scale of the density of the data points in the corresponding region is indicated on the right. Highlighted green circles are significantly oxidised (positive values) and reduced (negative values) peptides. (**d**) Relative oxygen consumption rate (OCR) in response to treatment with indicated concentrations of hydrogen peroxide. (**a-c**) Based on 4 independent experiments, single measurement; (**d**) mean (S.D.) of 3 independent experiments with 12 replicate measurements per treatment.

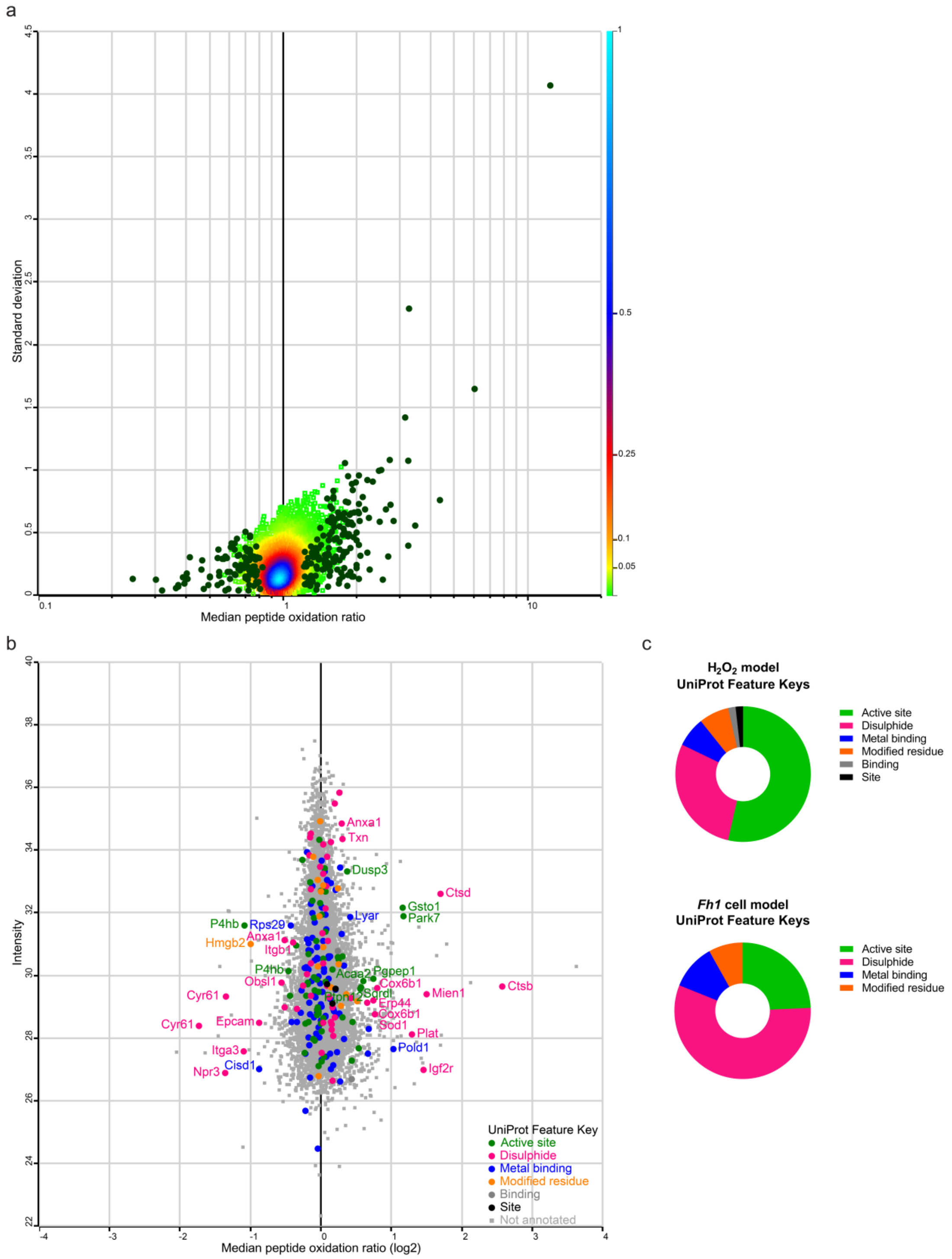

Supplementary Fig. 3. Chronic oxidative stress due to *Fh1* loss causes widespread protein oxidation. (**a**) Density scatterplot displaying median peptide oxidation ratios versus standard deviation in *Fh1*$^{-/-}$ cells compared to *Fh1*$^{fl/fl}$ cells. Every square represents a unique peptide; colour scale of the density of the data points in the corresponding region is indicated on the right. Highlighted green circles are significantly oxidised (positive values) and reduced (negative values)

peptides. (**b**) Scatterplot displaying log 2 median peptide oxidation ratios versus peptide intensity in *Fh1*$^{-/-}$ cells compared to *Fh1*$^{fl/fl}$ cells. Every symbol represents a unique peptide, with legend indicated. Coloured circles represent peptides of which the cysteine residue has a Feature Key annotation in the UniProt database; squares represent those without this annotation. Significantly oxidised (positive values) or reduced (negative values) cysteine peptides within the annotated group are highlighted with their corresponding gene name. (**c**) Comparison of cysteine residue function of the significantly oxidised or reduced cysteine peptides between the acute and chronic oxidative stress model, based on UniProt Feature Keys. (**a-c**) Based on 4 independent experiments, single measurement.

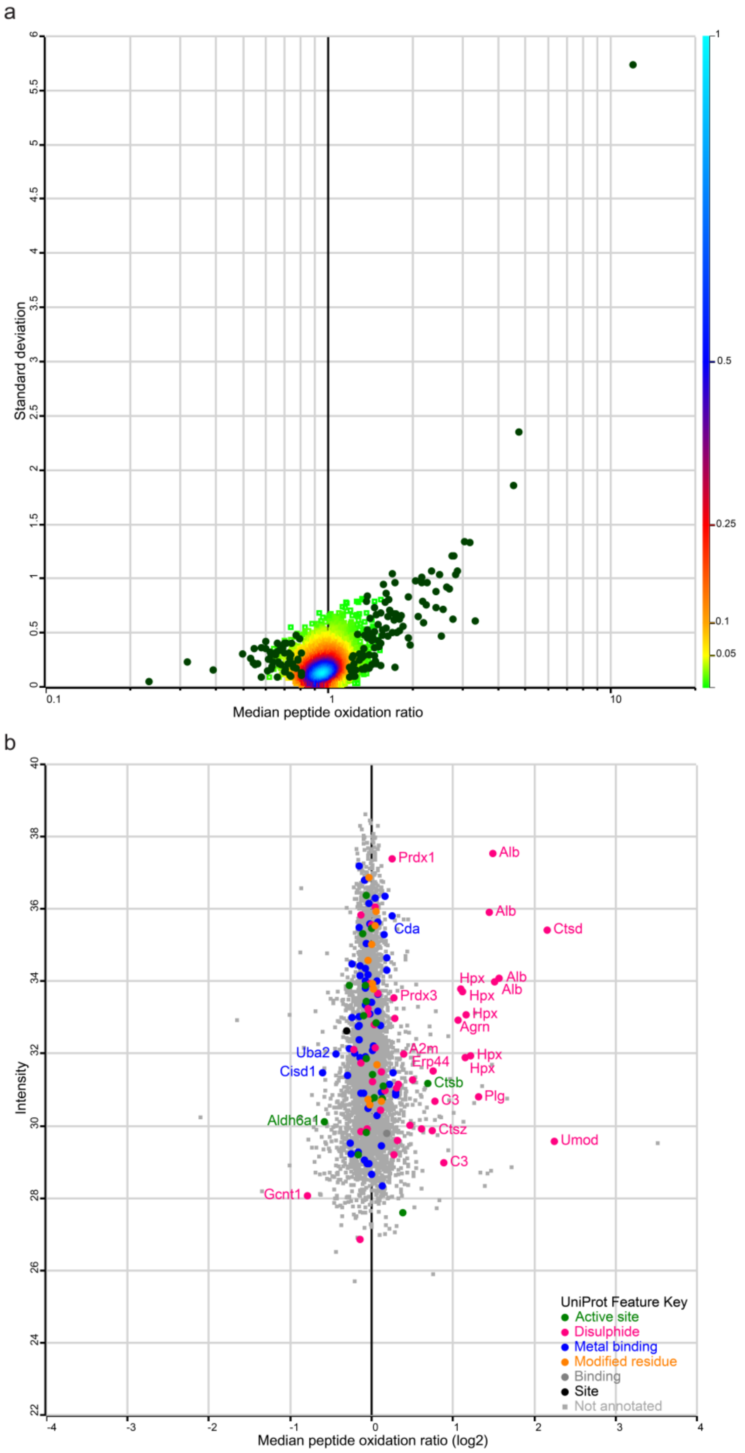

Supplementary Fig. 4. Analysis of mouse kidneys shows oxidation of proteins from various tissue compartments. (**a**) Density scatterplot displaying median peptide oxidation ratios versus standard deviation in *Fh1*$^{-/-}$ mouse kidney tissue compared to *Fh1*$^{fl/fl}$ kidney tissue. Every square represents a unique peptide; colour scale of the density of the data points in the corresponding region is indicated on the right. Highlighted green circles are significantly oxidised (positive values) and

reduced (negative values) peptides. (**b**) Scatterplot displaying log 2 median peptide oxidation ratios versus peptide intensity in *Fh1*$^{-/-}$ mouse kidney tissue compared to *Fh1*$^{fl/fl}$ kidney tissue. Every symbol represents a unique peptide, with legend indicated. Coloured circles represent peptides of which the cysteine residue has a Feature Key annotation in the UniProt database; squares represent those without this annotation. Significantly oxidised (positive values) or reduced (negative values) cysteine peptides within the annotated group are highlighted with their corresponding gene name. (**a-b**) Based on the comparison of 1 mouse per genotype, using 4 replicate tissue slices per mouse.

Supplementary Notes

Supplementary Note 1. Quality control strategy and statistical analysis of peptide oxidation ratios generated with SICyLIA

In the following, we describe the rationale underlying the multi-step quality control and statistical approach taken in our manuscript to select biologically meaningful redox-sensitive peptides. We also provide a comparison of methods for statistical analysis and their suitability for peptide oxidation analysis.

The aim of the SICyLIA methodology is to generate unbiased profiles of cysteine oxidation at a whole-proteome scale. As such, it can be used as a screening tool to identify oxidised proteins and modulated pathways in pathophysiological and other oxidative stress conditions in cells and tissues. It is important to note that biological cysteine oxidation is a transient event that is rapidly turned over. As such, peptide oxidation measurements are inherently variable. Therefore, an appropriate statistical test needs to be able to identify redox-sensitive peptides despite substantial variability in peptide oxidation measures.

In order to identify those peptides of interest that undergo the largest biological change in oxidation state, exemplified by the largest (positive and negative) oxidation ratios in the dataset, one could simply rank peptides by their oxidation ratio. However, highly abundant proteins and peptides display a more focused statistical spread than low abundant ones [1, 2]. This reflects the accuracy of the calculated peptide ratios: more intense peptides are generally quantified based on a higher number of quantification events. Thus, a hit selection strategy needs to take into account the influence of peptide intensity on measurement variation. For this reason, we opted for the Significance B algorithm in Perseus. This algorithm was specifically developed by Cox & Mann for the analysis of MS-based protein/peptide quantification using log protein/peptide ratios in SILAC experiments [1]. The algorithm calculates an outlier significance score for each data point that highlights how significant a change in that data point is as compared to all other data points. Importantly, the Significance B algorithm also considers the different intensities of the peptides when calculating significant outliers by creating intensity groups (bins) of equal occupancy for all peptides, over which the significance is calculated. The Significance B algorithm creates robust and asymmetrical estimates of the deviation from the main distribution by calculating the 15.87, 50 and 84.13 percentiles, which are right- and left-sided robust standard deviations. For normally distributed data, these are equal and meet the conventional definition of a standard deviation. These are then used to calculate a p-value to detect significant outlier ratios, defined as “Significance

B", which is the probability that a log-ratio of at least the magnitude observed is obtained (with the Benjamini-Hochberg correction for multiple hypothesis testing, FDR <5% [3]).

Given that the significance B algorithm compares one single data point against all other data points, and does not take into account multiple values of biological replicates, a summary statistic must be used for the replicate oxidation ratios of each peptide. We therefore opted to use the median oxidation ratio of the four replicate experiments. However, to take into account variability between replicates, stringent quality control (QC) measures were included to achieve accurate median cysteine oxidation ratios. First, only peptide oxidation ratios that were robustly quantified in at least three replicates were included. Additionally, the coefficient of variation (CV%) between replicates was used to filter out extreme outlier ratios. This was done by calculating upper fences (Q3 + 1.5IQR, where Q3 is the third quartile and IQR is the interquartile range) of the distribution of CVs for all median peptide oxidation ratios in each dataset and using these as cut-off. After QC, the median peptide and protein ratios between replicates were calculated and used for all analyses. In this way, we have minimised the effect of outliers on our analyses. In fact, after QC the overall variability across our datasets was low, with median CVs of 15.1 (H_2O_2 model), 17.7 (*Fh1* cell model), and 16.0 (*Fh1* tissue model).

Nevertheless, using median ratio values from replicates constructs a virtual one-sample. Thus, we assessed the robustness of statistical performance by conducting the Significance B test for the individual ratio values and evaluated whether the distribution of p-values obtained for each replicate was similar. Below, we show kernel density plots of the p-values for each replicate experiment for the three models. The grey line indicates the p-value of 0.05 used as threshold for significance in the study; we also report the mean and standard deviation (SD) of the distributions.

H_2O_2 model

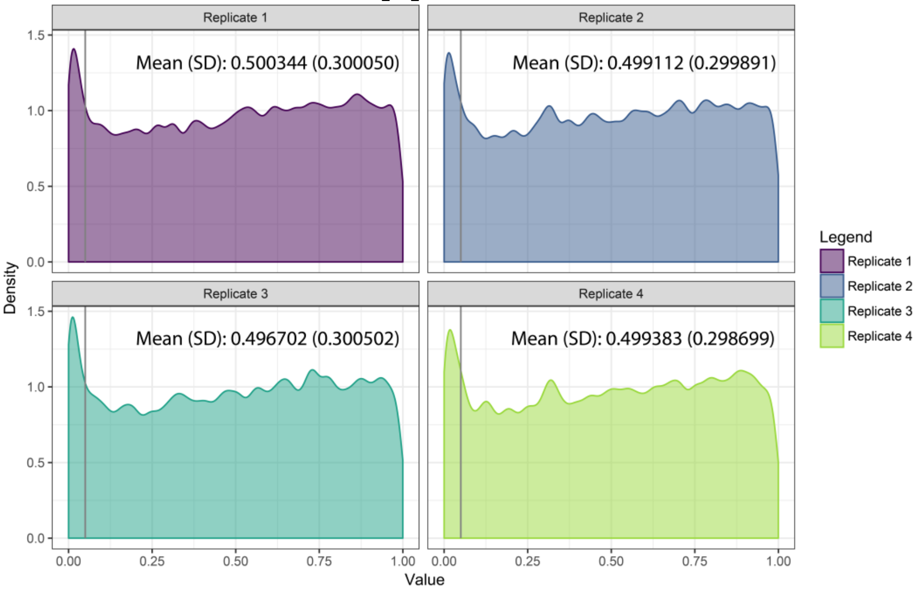

***Fh1* cell model**

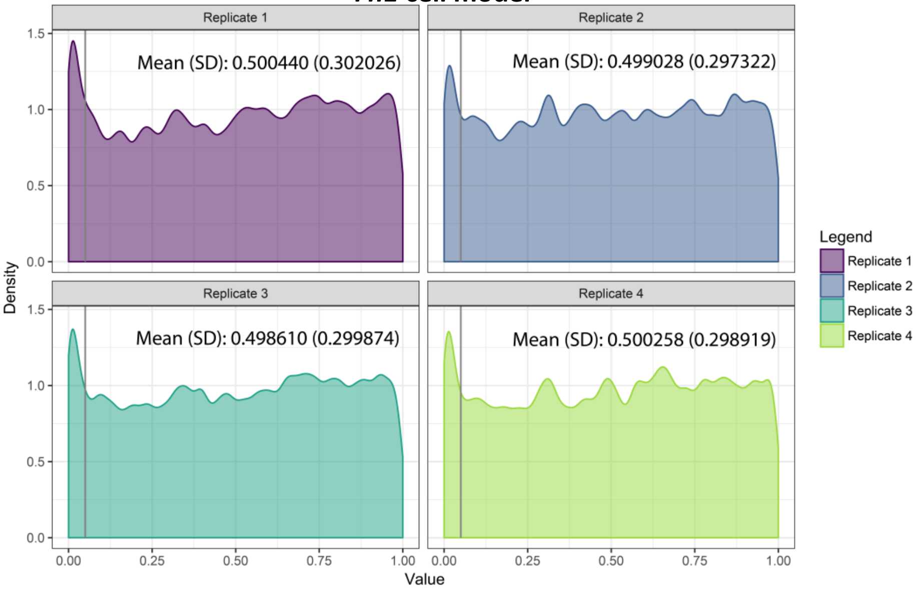

***Fh1* tissue model**

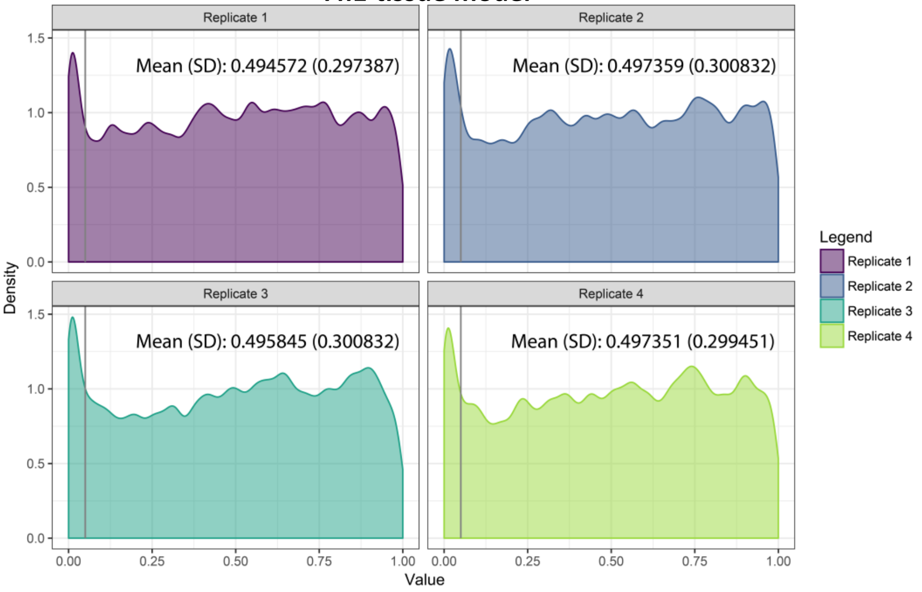

Supplementary Fig. 5. Kernel density plots displaying the distribution of p-values obtained using the Significance B algorithm for each individual replicate in the H_2O_2 model (**top**), *Fh1* cell model (**middle**), and *Fh1* tissue model (**bottom**). Mean and standard deviation (SD) of the individual distributions are displayed.

As can be appreciated, the kernel density plots and standard deviations of the p-values show a high degree of similarity for the four replicates of each experimental model. Thus, we decided to use the median ratio value for assessment of statistical significance. Importantly, using the median peptide oxidation ratio also ensures that the peptides that will be identified as significant are those that consistently change their oxidation state in the same direction (as peptides that are reduced (negative value) in some, but oxidised (positive value) in other replicates will have a median oxidation ratio close to 0 and will not be considered significant). This is an important consideration when comparing the median value and the individual values for each replicate, as it explains that they are not necessarily expected to be the same: cysteine residues can inherently exist in both an oxidised and reduced state. In the current study, we chose to limit our analysis to those peptides that are consistently more oxidised or more reduced in replicate experiments. Nevertheless, to increase transparency the individual peptide oxidation ratios and their Significance B values are reported for each replicate in the Supplementary Data as well.

Another statistical test that can be used to analyse data that contain biological replicates is the t-test. In the current study, to determine significant changes in oxidation ratios, one would use a 1-sample t-test under the null hypothesis that the log2 oxidation ratio is 0 (with 0 meaning no change in oxidation state). Compared to the Significance B approach described above, the 1-sample t-test does not require a multi-step filtering approach because the variability between replicates is already taken into account for the calculation of the p-values. Thus, we decided to compare the two methods and assess their suitability for our study. Shown below are the results for the dataset of hydrogen peroxide-treated cells.

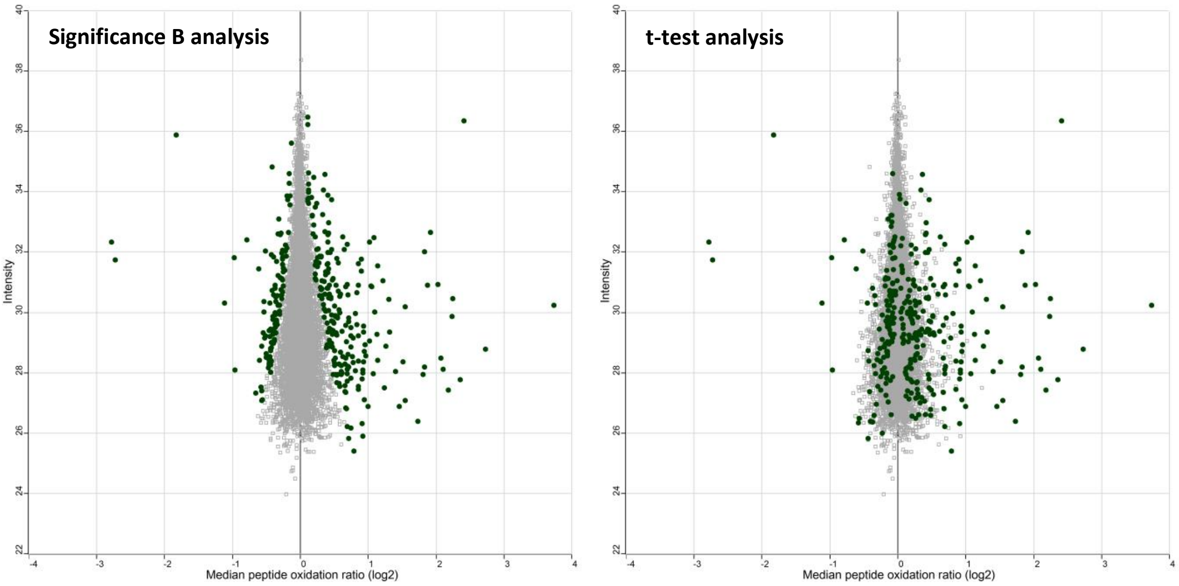

Supplementary Fig. 6. Scatterplots displaying log 2 median peptide oxidation ratios versus peptide intensity in *Fh1*$^{fl/fl}$ cells treated with 500 µM hydrogen peroxide for 15 minutes compared to untreated cells. Every square represents a unique peptide; highlighted green circles are significantly oxidised (positive values) or reduced (negative values) peptides using either the Significance B algorithm (**left**) or t-test (**right**).

Both figures show scatterplots of the median oxidation ratios of all peptides included in the analysis, plotted against their intensity. Significant hits are highlighted in green. The left panel shows “Significance B” analysis (333 hits), the right panel shows the t-test results ($p<0.05$; 327 hits). It can be appreciated that peptides with oxidation ratios that change most dramatically after treatment (both positively and negatively) are considered significant using both analyses. In total, 117 hits were significant using both tests. However, we found that the t-test favours many peptides with small ratio values, which are found in the middle of the distribution. These peptides undergo only a marginal change in oxidation state, which is exemplified by an oxidation ratio close to 0, but have low experimental variability and therefore pass significance using the t-test. As the SICyLIA methodology aims to identify the most redox-reactive peptides, this is undesirable.

We next set out to determine why peptides that undergo larger changes in oxidation state (and are thus most interesting from a biological perspective) do not pass significance using the t-test. We observed that in our data, variability is inherent to those peptides that undergo the largest biological change in oxidation, or those that are of lower abundance. This is more clearly demonstrated in the following figures:

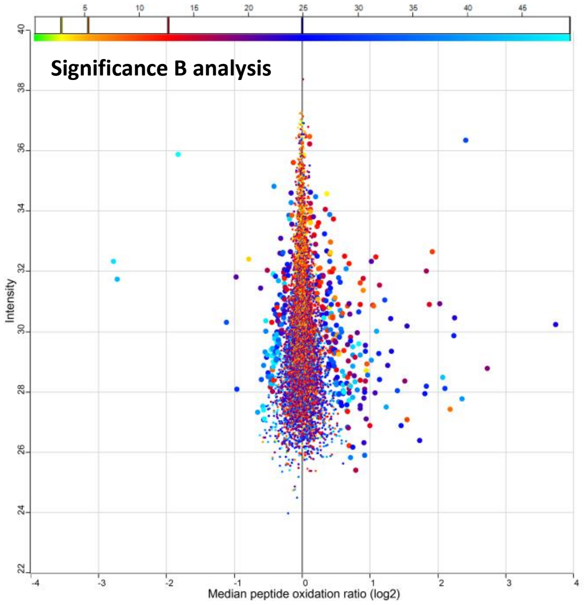

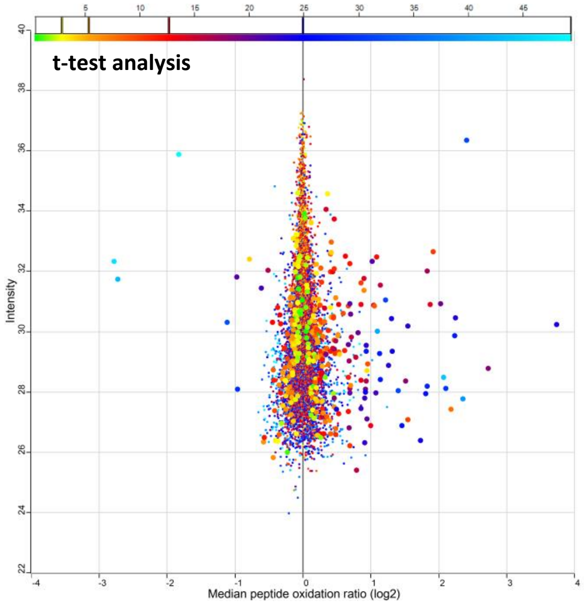

Supplementary Fig. 7. Scatterplots displaying log 2 median peptide oxidation ratios versus peptide intensity in *Fh1*$^{fl/fl}$ cells treated with 500 µM hydrogen peroxide for 15 minutes compared to untreated cells. Every square represents a unique peptide. Peptides are coloured by the value of the coefficient of variation between the replicate experiments; colour scale of the data points is indicated above. Highlighted circles are significantly oxidised (positive values) or reduced (negative values) peptides using either the Significance B algorithm (**left**) or t-test (**right**).

Both figures show scatterplots of oxidation ratios of all peptides included in the analysis, plotted against their intensity. Peptides are coloured by the value of the CV% between the replicate experiments, scale indicated above. Significant hits using Significance B (left) and the t-test (right) are highlighted by large filled circle symbols. As can be appreciated, the CV% increases for peptides with larger oxidation ratios and also those of lower intensity (blue colours). Clearly, the t-test favours those peptides with low CV% (green, yellow, orange colours). This is further exemplified by the following figure, which shows the oxidation ratio of all peptides identified plotted against their CV%. Highlighted in green are the hits that pass significance using t-test analysis.

t-test analysis

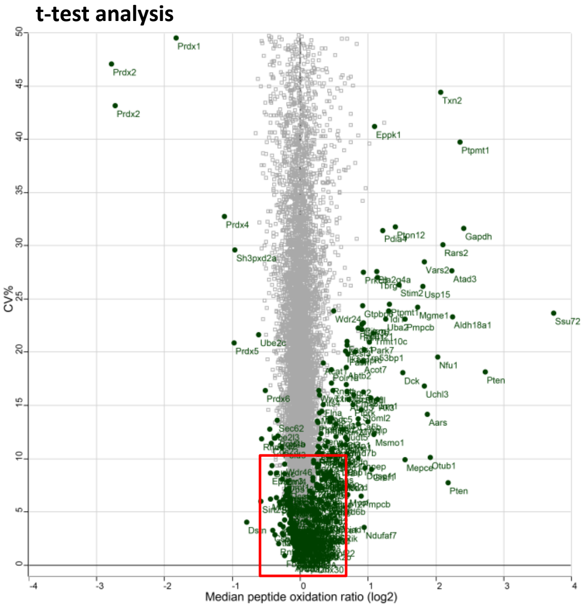

Supplementary Fig. 8. Scatterplots displaying log 2 median peptide oxidation ratios versus their coefficient of variation in *Fh1*$^{fl/fl}$ cells treated with 500 µM hydrogen peroxide for 15 minutes compared to untreated cells. Every square represents a unique peptide. Highlighted circles are significantly oxidised (positive values) or reduced (negative values) peptides using the t-test, with the gene names of corresponding proteins displayed.

This figure clearly shows that variation is the main parameter that determines whether a peptide will be considered significant using the t-test, despite many of them having an oxidation ratio close to 0 and thus marginal changes in oxidation state (highlighted in the figure above). As the aim of the SICyLIA methodology is to identify peptides that are meaningfully redox-reactive, and thus those with a larger positive or negative oxidation ratio, we concluded that the t-test was not suitable for the aim of our study. In contrast, the significance B algorithm selects those peptides that have undergone a significantly larger change in oxidation state compared to all other peptides of similar intensity identified, and thus, identifies the most redox-sensitive peptides in the dataset.

The above analysis shows that variation in the quantified peptide oxidation ratios is inherent for peptides that undergo the largest biological change in oxidation, or those that are of lower abundance. However, we found that this increased variation does not reflect a lack of biological significance. This can be illustrated by highlighting some top hits identified using SICyLIA: these are *bona fide* redox targets of known function, which have been extensively shown to play a role in cellular redox biology by researchers in the field (GAPDH [4], PARK7 [5-7], PTEN [8], Thioredoxin 2 [9], and the Peroxiredoxin family members [10]):

Gene name	Peptide oxidation ratio	CV%	Cysteine biological function
Gapdh	2.4	32	Active site
Park7	1.0	21	Active site
Pten	2.7	18	Active site
Txn2	2.1	44	Disulphide bond
Prdx1	-1.8	49	Disulphide bond
Prdx2	-2.8	47	Disulphide bond
Prdx2	-2.7	43	Disulphide bond
Prdx4	-1.1	33	Disulphide bond
Prdx5	-1.0	21	Disulphide bond
Prdx6	-0.5	16	Active site

There is a wide range of variabilities for these peptides, with all showing relatively high CV% as compared to the median CV% of all identified peptides (which is 15.1 in this dataset). All of these peptides pass significance using t-test analysis too, driven by their large change in oxidation ratio. However, in addition to these cysteine peptides known to be extremely reactive, identifying peptides that have milder changes in oxidation states is of great interest too, because they can also be biologically relevant redox-sensitive proteins. This is a key aim of the SICyLIA methodology: due to its unprecedented depth compared to previous methods, it can be used as a screening tool to identify novel redox-sensitive proteins besides the highly reactive redox sensors that are already well-characterised. The more mildly reactive peptides are those that can be seen at the edges of the data distribution, as their reactivity stands out from the total amount of peptides in the dataset, of which the majority (>96%) is not redox-regulated. These peptides are identified using the Significance B algorithm. However, due to the inherent variability of biological cysteine oxidation they do not pass the t-test.

It is important to remember that the oxidation ratios are measured at the peptide level. Such measurements are inherently more variable than measurements at the protein level, which are the resultant of tens of individual peptide measurements. This variation at the peptide oxidation level does not, however, indicate poor biological reproducibility in our methodology. As detecting peptide cysteine oxidation at whole proteome scale is a matter of locating small differences within big background noise, we therefore recommend applying multi-step filtering rather than relying solely on a t-test to select results with biological relevance.

Finally, another strategy is to combine the Significance B approach with the 1-sample t-test and include only those peptides that pass both tests in downstream analyses. This would mitigate the issue that the t-test selects many peptides that are well-identified but do not actually undergo a substantial redox change, as the subsequent Significance B filter will select the most redox-sensitive peptides that have passed significance using the t-test. We applied this strategy to all datasets, and it achieved the same global results obtained with the Significance B analysis alone, thus further supporting the validity of using the Significance B approach. This combined strategy may be a useful approach if the aim is to select only highly redox-reactive peptides that undergo large changes in oxidation state, but less so if the aim is to identify more mildly redox-sensitive peptides.

In conclusion, to be able to capture cysteine oxidation at the global proteome level, without limiting our study to highly redox-reactive proteins, we decided to use the Significance B algorithm, knowing that it remains an important consideration that biological replicates are not fully considered when assessing significance using this test. As variation between replicates is best interpreted at the single peptide level, we have provided the CV% and standard deviation between replicate experiments for each peptide in the Supplementary Data to aid the selection of the most meaningful hits from our study.

Supplementary References

1. Bantscheff, M., Schirle, M., Sweetman, G., Rick, J. & Kuster, B. Quantitative mass spectrometry in proteomics: a critical review. *Anal. Bioanal. Chem.* **389,** 1017-1031 (2007).
2. Cox, J. & Mann, M. MaxQuant enables high peptide identification rates, individualized p.p.b.-range mass accuracies and proteome-wide protein quantification. *Nat. Biotechnol.* **26,** 1367-1372 (2008).
3. Benjamini, Y. & Hochberg, Y. Controlling the False Discovery Rate: A Practical and Powerful Approach to Multiple Testing. *J. R. Stat. Soc. Series B Stat. Methodol.* **57**: 289-300 (1995).
4. Peralta, D. et al. A proton relay enhances H2O2 sensitivity of GAPDH to facilitate metabolic adaptation. *Nat. Chem. Biol.* **11,** 156-163 (2015).
5. Kim, R.H. et al. DJ-1, a novel regulator of the tumor suppressor PTEN. *Cancer Cell* **7,** 263-273 (2005).
6. Taira, T., Saito, Y., Niki, T., Iguchi-Ariga, S.M.M., Takahashi, K. & Ariga, H. DJ-1 has a role in antioxidative stress to prevent cell death. *EMBO Rep.* **5,** 213-218 (2004).
7. Clements, C.M., McNally, R.S., Conti, B.J., Mak, T.W. & Ting, J.P.Y. DJ-1, a cancer- and Parkinson's disease-associated protein, stabilizes the antioxidant transcriptional master regulator Nrf2. *Proc. Natl. Acad. Sci. U.S.A.* **103,** 15091-15096 (2006).
8. Lee, S.-R., Yang, K.-S., Kwon, J., Lee, C., Jeong, W. & Rhee, S.G. Reversible Inactivation of the Tumor Suppressor PTEN by H2O2. *J. Biol. Chem.* **277,** 20336-20342 (2002).
9. Spyrou, G., Enmark, E., Miranda-Vizuete, A., Gustafsson, J. Cloning and Expression of a Novel Mammalian Thioredoxin. *J. Biol. Chem.* **272,** 2936-2941 (1997).
10. Wood, Z.A., Schröder, E., Robin Harris, J., Poole, L.B. Structure, mechanism and regulation of peroxiredoxins. *Trends Biochem. Sci.* **28,** 32-40 (2003).
