# Supplementary material for: Proteome-wide analysis of cysteine oxidation reveals metabolic sensitivity to redox stress
Source: Nat Commun. 2018 Apr 20;9:1581. doi: 10.1038/s41467-018-04003-3 (PMC5910380; doi:10.1038/s41467-018-04003-3)
Supplement: Supplementary file 3 — Description of Additional Supplementary files [file 41467_2018_4003_MOESM3_ESM.pdf]

Description of Additional Supplementary Files

File Name: Supplementary Data 1

Description: SICyLIA proteomic results of all cysteine-containing peptides identified in *Fh1*$^{fl/fl}$ cells treated with 500 µM hydrogen peroxide for 15 minutes compared to untreated cells, corresponding to Fig. 4 and Supplementary Fig. 2. Based on 4 independent experiments, single measurement. A Significance B value of 0.00E+00 indicates highly significant.

File Name: Supplementary Data 2

Description: SICyLIA proteomic results of all cysteine-containing peptides identified in *Fh1*$^{-/-}$ compared to *Fh1*$^{fl/fl}$ cells, corresponding to Fig. 7 and Supplementary Fig. 3. Based on 4 independent experiments, single measurement.

File Name: Supplementary Data 3

Description: SICyLIA proteomic results of all cysteine-containing peptides identified in *Fh1*$^{-/-}$ compared to *Fh1*$^{fl/fl}$ mouse kidney tissues, corresponding to Fig. 8 and Supplementary Fig. 4. Based on the comparison of 1 mouse per genotype, using 4 replicate tissue slices per mouse.
